# The relationship between age, axial length and retinal nerve fiber layer thickness in the normal elderly population in Taiwan: The Chiayi eye study in Taiwan

**DOI:** 10.1371/journal.pone.0194116

**Published:** 2018-03-09

**Authors:** Chau-Yin Chen, Evelyn Jou-Chen Huang, Chien-Neng Kuo, Pei-Lun Wu, Ching-Lung Chen, Pei-Chen Wu, Shin-Hua Wu, Yin-Chi King, Chien-Hsiung Lai

**Affiliations:** 1 Department of Ophthalmology, Chang Gung Memorial Hospital, Chiayi, Taiwan; 2 College of Medicine, Chang Gung University, Tao-Yuan, Taiwan; 3 Department of Ophthalmology, China Medical University Beigang Hospital, Yunlin, Taiwan; 4 Department of Nursing, Chang Gung University of Science and Technology, Chiayi, Taiwan; Charite Universitatsmedizin Berlin, GERMANY

## Abstract

**Aims:**

To interpret how the thickness of the peripapillary retinal nerve fiber layer (RNFL) changes with increasing age, axial length, or anterior chamber depth as measured by spectral domain optical coherence tomography (OCT) in the normal elderly population in Taiwan.

**Methods:**

A total of 82 volunteers (143 eyes) were enrolled. Generalized estimating equations were used to evaluate the correlation.

**Results:**

The RNFL was significantly thinner in the superonasal (p = 0.004), inferotemporal (p = 0.046), and temporolower (p = 0.009) segments with age. The same trend was also observed in the superotemporal (p = 0.330) segment, although it was not statistically significant. The global RNFL thickness decreased by 4.97 μm per decade (β = -0.497; p = 0.021), and thinning was significant in the superonasal (-9.90 μm per decade, p < 0.001) and temporolower (-6.78 μm per decade, p < 0.001) segments; the same trend showed borderline significance in the superotemporal (-6.96 μm per decade, p = 0.073) and inferotemporal (-7.23 μm per decade, p = 0.059) segments. In eyes with longer axial length, the RNFLs significantly decreased in the non-temporal segments. Global RNFL thickness decreased by 3.086 μm for each additional millimeter of axial length (β = -3.086; p < 0.001).

**Conclusions:**

Changes in RNFL thickness were correlated with age in the superonasal, superotemporal, inferotemporal, and temporolower segments, and were correlated with axial length in the non-temporal segments. Anterior chamber depth was not correlated with RNFL thickness.

## Introduction

Glaucoma and other optic neuropathies are a group of diseases manifesting with progressive retinal ganglion cell and axon loss, which can result in visual disturbance and visual field defects. Significant correlations have been described between morphological changes of the optic disc and functional changes in vision. It has been suggested that morphological abnormalities can develop early before any functional loss is detected [1, 2]. Therefore, subjective clinical examination of the optic nerve head (ONH) allows for assessment of early diagnosis before functional defects manifest, and for monitoring of the structural changes associated with disease progression [3–5].

Since the introduction of optical coherence tomography (OCT), a non-invasive technique that allows cross-sectional tomographic imaging of the retina and optic nerve, the ONH and the thickness of the retinal nerve fiber layer (RNFL) can now be measured in vivo. The peripapillary RNFL thickness measurements acquired using OCT have been used for the detection and monitoring of glaucoma and other optic neuropathies [1, 2, 4, 5]. In addition, the use of OCT has extended beyond RNFL measurements for glaucoma, and has also been used in the investigation of cognitive impairment and neurodegenerative disorders such as Alzheimer’s disease (AD) and multiple sclerosis (MS) [6–8]. Visual disturbance can be a symptom of AD or MS, reflecting neuronal damage in the visual pathway. In a review, Jones-Odeh highlighted the association between these disorders and ocular structure (RNFL and ganglion cell layer), and examined their usefulness as biomarkers of neurodegeneration. The average RNFL thickness loss in patients with AD is 11 μm; in patients with MS, it is 7 μm. However, it is unclear whether the main ocular biomarker of RNFL thinning investigated for AD and MS is secondary to cortical loss (retrograde damage), or if the same disease process affects both the brain and the retina [8, 9].

The degree of cataract formation could affect the signal quality in OCT. A study showed that OCT-measured RNFL thickness had increased one month after cataract surgery, which may have been caused by an improvement in OCT signal quality [10]. Other studies have also revealed that dense cataracts can cause the peripapillary RNFL to appear thinner on OCT scan due to changing signal quality. Therefore, thinning of the peripapillary RNFL, which indicates glaucomatous progression, may simply be an artifact of a severe cataract when measured using OCT [11].

OCT-measured RNFL thickness is also subject to variability due to many factors, such as age, gender, ethnicity, axial length, and optic disc area. There is yet no consensus as to which factors affect the measurement of RNFL thickness with OCT in normal subjects. It is highly likely that these factors affect the RNFL thickness differently, and hence affect diagnosis and monitoring in diseased conditions [12].

OCT was first described in 1991 by Huang et al. [13] and has since undergone many significant technological advances. Currently, each OCT machine has its own database of age-matched normal values used to interpret RNFL thickness measurements. However, most normative databases comprise measurements from subjects of European descent. Therefore, it is important that researchers ascertain normal RNFL thickness values in other races, in our case, the Taiwanese. By doing so, clinicians will be able to distinguish normal from pathological changes more clearly.

For these reasons, we aimed to interpret the thickness of the peripapillary RNFL measured using spectral domain OCT (SD-OCT) in the normal elderly population in Taiwan, as well as to establish normative data regarding how RNFL thickness changes with increasing age, axial length, or anterior chamber depth. In order to ensure a more rigorous design, our study excluded participants with neurodegenerative diseases or cognitive impairment (such as AD and MS), cataracts identified as worse than moderate, and patients who had undergone cataract surgery.

## Materials and methods

### Statement of ethics

This study adhered to the tenets of the Declaration of Helsinki and was approved by the Institutional Review Board of Chang Gung Memorial Hospital, Chiayi, Taiwan. All participants provided written informed consent after researchers had verbally explained the study to them, and we followed all applicable institutional and governmental regulations concerning ethics in human research.

### Study population

Puzih is a coastal village in south-western Taiwan located at latitude 23°N. The population of the village has a higher mean age than that of other cities in Taiwan; moreover, mean income is lower, and fewer inhabitants receive adequate eye care. Most residents of Puzih work outdoors as farmers, fishermen, or brine evaporators. We conducted a cross-sectional population-based survey in Puzih from March 2012 to March 2014. When our study began, there were 6786 individuals aged ≥ 65 years in the village [14]; we used residents’ identification numbers to randomly invite 700 of these residents to participate in our study. Of the 700 invited individuals, 132 came to our hospital for ocular examinations. After analyzing the results of the examination, 50 participants who did not meet the study criteria were excluded. Finally, 82 participants (11.7%) with 143 normal physical eyes were ultimately enrolled. Furthermore, our study cohort represented only 1.208% of the elderly population of Puzih.

### Exclusion and inclusion criteria

In order to strictly collect data on the normal eyes of the elderly population, any participant with ocular disease that affect RNFL measurement by OCT were excluded from our study. We excluded eyes with a history of corneal scarring, intraocular disease (e.g., uveitis, trauma, diabetic retinopathy, retinal vessel occlusion disease, macular atrophy, or age-related macular degeneration), more than moderate cataracts, diseases that can affect the parapapillary area (e.g., coloboma, large parapapillary atrophy or ambiguous disc disease, optic neuropathy, diffuse atrophy, staphyloma, cognitive impairment or neurodegenerative disorder such as AD and MS), and any ocular surgery (including cataract extraction). To eliminate interference from other diseases, such as glaucoma, other optic disc diseases, cognitive impairment, or neurodegenerative disorders, we only included patients with a normal visual field presentation.

### Procedure

All participants underwent a complete ophthalmologic examination, including manifest refraction, slit lamp examination, standard automated perimetry, best corrected visual acuity (by Snellen-chart), anterior chamber depth, axial length, and intraocular pressure. After full pupil dilation, each eye was imaged using a color fundus camera (CF-60UD^™^, Canon Inc., Tokyo, Japan) and OCT. Patients’ age, sex, and height were also recorded.

### Optical coherence tomography

We performed SD-OCT using a Stratus^™^ OCT 3000 (Carl Zeiss, Oberkochen, Germany). In this scanner, a super luminescent diode laser with a center wavelength of 840 nm serves as a broadband light source to generate back reflections from different intraretinal depths, which are represented by different wavelengths. The RNFL thickness was measured using axial scans (pixels) in an area of the optic disc measuring 6 mm × 6 mm. The thickness at each pixel was measured, and a map was generated. A calculation circle with a diameter of 3.46 mm was then automatically placed around the optic disc. To display the RNFL thickness distribution around the optic disc, the thickness data of 8 segments (45° each) were averaged; these 8 segments were grouped into 2 sectors (superior half and inferior half, with 0° corresponding to the temporal, horizontal position).

### Statistics

In statistics, a generalized estimating equation (GEE) is used to estimate the parameters of a generalized linear model with a possible unknown correlation between outcomes. It is also a better estimating method for clustered or multi-level data. In our study, two eyes were nested under each hierarchy (participant) with one age; it was necessary to consider the differences caused by each hierarchy. The use of the GEE at this time considered the fact that the individual level units (eyes) under each hierarchy (each individual, participant) were interdependent. The focus of the GEE was on estimating the average response over the population (population-averaged effects) rather than the regression parameters that would enable prediction of the effect of changing one or more covariates on a given individual.

## Results

### Study population and patient characteristics

Of the 264 eyes in 132 people who participated in the study, 121 eyes were excluded from the analysis because their baseline characteristics in OCT had been changed following the diseases listed in Table 1. Three eyes with dense cataract and four eyes with corneal opacity that hindered the penetration of light from the OCT instrument that resulted in OCT study failure. Three eyes with a history of RVO (retinal vessel occlusion) showed focal retinal laser spots within the nourished area of retinal vein thrombosis that made those areas lose of retinal nerve fibers. Other three eyes with retinal scars of unknown etiology, which were thought to affect the results of RNFL thickness measurement were excluded. One participant who suffered from binocular vision loss with presenting binocular pale discs and visual field defects, was then referred to the Neuro-physician for suspecting a brain lesion. After eliminating those who met the exclusion criteria, the remaining eyes must meet the normal visual field. Finally, total 143 eyes in 82 participants were included in the analysis.

**Table 1:** The number of eyes and participants that met the criteria for inclusion or exclusion.

	Number of eyes (persons)
**Exclusion criteria:**	Total 121 eyes
Pseudophakia	64
Glaucoma	37
Diabetic retinopathy	8
Age-related macular degeneration	18
Dense cataract	8
Corneal scar or degeneration	4
Retinal vessel occlusion disease	3
Other retinal scar	3
Retinitis pigmentosa	2 (in 1 person)
High myopia peripapillary atrophy	3 (in 2 persons)
Other optic neuropathy	2 (in 2 persons)
Dementia	2 (in 1 person)
Brain lesion	2 (in 1 person)
**Inclusion criteria:** Remaining eyes after selecting by exclusion criteria, that had normal visual field.	Total 143 eyes (in 82 persons)

Of total 264 eyes from 132 participants for the examination, 121 eyes met the exclusion criteria were excluded from the study. Finally, 143 eyes of 82 participants were included for statistical analysis.

Table 2 shows the demographic characteristics of the patients. The subjects had an average age of 69.85 years. Abnormal eyes were excluded according to the aforementioned criteria. Ultimately, 143 eyes were included in the analysis. These had a mean axial length of 23.43 ± 0.98 mm and a mean RNFL thickness of 102.84 ± 9.21 μm.

**Table 2:** Characteristics of the 82 study participants and mean RNFL thicknesses of different segments.

Number of patients	82
Number of eyes	143
Mean age (years)	69.85
mean axial length ± SD (mm)	23.43 ± 0.98
**Mean RNFL thicknesses of different segments**	**Mean ±SD (μm)**
NU	82.64 ± 11.80
NL	74.33 ± 11.04
IN	122.94 ± 21.94
IT	141.66 ± 19.77
TL	76.74 ± 12.02
TU	83.77 ± 12.36
ST	129.15 ± 17.93
SN	109.67 ± 15.00
SAVG	101.32 ± 9.54
IAVG	104.00 ± 11.44
AVGThick	102.84 ± 9.21

NU: nasoupper, NL: nasolower, IN: inferonasal, IT: inferotemporal, TL: temporolower, TU: temporoupper, ST: superotemporal, SN: superonasal, SAVG: average RNFL thickness of superior half, IAVG: average RNFL thickness of inferior half, AVGThick: average RNFL thickness globally, SD: standard deviation

### Mean RNFL thickness of different segments of the eyes

The RNFL thickness of different peripapillary segments is detailed in Table 1, the higher values occurred in the inferotemporal (141.66 ± 19.88 μm) and superotemporal segments (129.15 ± 17.93 μm), while the lower values were in the nasal (nasoupper: 82.64 ± 11.80 μm, nasolower: 74.33 ±11.04 μm) and temporal segments (temporoupper: 83.77 ± 12.36 μm, temporolower: 76.74 ± 12.02 μm).

### The correlation between RNFL thickness and age

Older participants tended to have thinner RNFLs (Table 3). The global average values of RNFL thickness were 103.62 ± 8.29 μm in those aged less than 70 years, 101.63 ± 9.51 μm in those aged between 70 and 79 years, and 96.17 ± 12.30 μm in those aged between 80 and 89 years. The trend towards thinner RNFL with increasing age had borderline significance (p = 0.055); furthermore, it occurred in both the superior and inferior halves of the peripapillary retina nerve fiber layer, although it was not significant in either group (p = 0.114 and 0.107, respectively). Specifically, in the superior quadrant, the average RNFL thickness was 102.18 ± 8.77 μm in those aged less than 70 years, 99.56 ± 9.87 μm in those aged between 70 and 79 years, and 96.45 ± 11.92 μm in those aged between 80 and 89 years. In the inferior half, the average RNFL thickness was 104.60 ± 9.90 μm in those aged less than 70 years, 103.39 ± 13.16 μm in those aged between 70 and 79 years, and 95.88 ± 13.78 μm in those aged between 80 and 89 years.

**Table 3:** Mean RNFL thickness of the different segments among the three age groups.

Segment	Age < 70 (n = 92) RNFL (mean ± SD) (μm)	Age 70–79 (n = 43) RNFL (mean ± SD) (μm)	Age 80–89 (n = 8) RNFL (mean ± SD) (μm)	p-value
NU	82.87 ± 10.95	81.28 ± 13.28	81.75 ± 7.13	0.750
NL	73.29 ± 10.02	75.14 ± 12.53	76.25 ± 9.35	0.545
IN	122.82 ± 19.70	123.37 ± 24.64	113.88 ± 28.28	0.513
IT	143.33 ± 16.84	140.07 ± 24.12	125.75 ± 18.74	0.046
TL	78.67 ± 12.48	74.79 ± 10.20	66.50 ± 9.64	0.009
TU	83.75 ± 12.60	84.81 ± 11.59	83.13 ± 11.80	0.875
ST	129.92 ± 16.27	127.44 ± 18.28	121.00 ± 25.46	0.330
SN	112.08 ± 14.03	104.74 ± 14.34	100.13 ± 13.80	0.004
superior half	102.18 ± 8.77	99.56 ± 9.87	96.45 ± 9.51	0.114
inferior half	104.60 ± 9.90	103.39 ± 13.16	95.88 ± 9.51	0.107
Global mean (μm)	103.62 ± 8.29	101.63 ± 9.51	96.17 ± 9.51	0.055

n: number of eyes, RNFL: retinal nerve fiber layer, SD: standard deviation, NU: nasoupper, NL: nasolower, IN: inferonasal, IT: inferotemporal, TL: temporolower, TU: temporoupper, ST: superotemporal, SN: superonasal

Table 3 also shows the mean RNFL thickness of the peripapillary segments in each age group. The RNFL was significantly thinner in the superonasal (p = 0.004), inferotemporal (p = 0.046), and temporolower (p = 0.009) segments in older participants. The same trend was observed in the superotemporal (p = 0.330) segment but was not statistically significant. No differences in RNFL thickness were observed in the other three segments (nasoupper, inferonasal, and temporoupper) among the three age groups. We observed the opposite trend in the nasolower segment, although it was not significant (p = 0.545).

Table 4 shows the differences in RNFL thickness among the three age groups in the different peripapillary segments. The global RNFL thickness decreased with age by 4.97 μm per decade (β = -0.497; p = 0.021), and the segmental RNFL thickness significantly decreased in the superonasal (-9.90 μm per decade, p = 0.001) and temporolower (-6.78 μm per decade, p = 0.001) segments; the same trend showed borderline significance in the superotemporal (-6.96 μm per decade, p = 0.073) and inferotemporal (-7.23 μm per decade, p = 0.059) segments. Similar trends occurred in all other segments with non-significant negative correlation except the temporoupper segment, where there was a non-significant positive correlation (p = 0.733).

**Table 4:** Differences in RNFL thickness of the different segments among the three age groups.

RNFL change with age (per decade)			
Segment	Adjusted difference		
	*β*	SD	p-value
NU	-0.261	0.205	0.204
NL	-0.022	0.213	0.918
IN	-0.538	0.555	0.332
IT	-0.723	0.382	0.059
TL	-0.678	0.209	0.001
TU	0.075	0.22	0.733
ST	-0.696	0.388	0.073
SN	-0.99	0.285	0.001
Global RNFL	-0.497	0.215	0.021

RNFL: retinal nerve fiber layer, SD: standard deviation, NU: nasoupper, NL: nasolower, IN: inferonasal, IT: inferotemporal, TL: temporolower, TU: temporoupper, ST: superotemporal, SN: superonasal

### Inverse correlation between RNFL thickness and axial length

As shown in Table 5, the mean axial length was 23.43 ± 0.98 mm. Axial length was significantly inversely correlated with mean global RNFL thickness (r = -0.282; p = 0.001). Eyes with a greater axial length tended to have a thinner RNFL. Furthermore, axial length was inversely correlated with RNFL thickness in both the superior half (r = -0.209; p = 0.013) and the inferior half (r = -0.257; p = 0.002). Anterior chamber depth was not correlated with RNFL thickness.

**Table 5:** The inverse correlation between RNFL thickness and axial length.

	Mean RNFL of each segment (μm)	coefficient (r)	p-value
Axial length	Global mean	-0.282	0.001
	Mean of superior half	-0.209	0.013
	Mean of inferior half	-0.257	0.002
Anterior chamber depth	Global mean	-0.136	0.105
	Mean of superior half	-0.062	0.470
	Mean of inferior half	-0.141	0.097

RNFL: retinal nerve fiber layer

Table 6 shows the analysis of the β values in the different segments. Global RNFL thickness decreased by 3.086 μm for each additional millimeter of axial length (β = -3.086; p < 0.001). In eyes with a longer axial length, the RNFL of non-temporal segments significantly decreased (nasoupper, nasolower, inferonasal, superonasal, superotemporal; p < 0.05 in all segments). This phenomenon did not occur in the temporal area (temporolower and temporoupper; p = 0.116 and 0.523, respectively). Furthermore, the same reverse pattern trend was also found in the inferotemporal segment, but the p-value was borderline (p = 0.06). We concluded that increased axial length was significantly correlated with thinner RNFL in all except the temporal segments.

**Table 6:** Variations in RNFL thickness of different segments with axial length.

RNFL with axial length			
Segments	Adjusted difference		
	*β*	SD	p-value
NU	-2.110	1.063	0.047
NL	-2.864	0.995	0.004
IN	-6.852	1.905	< 0.001
IT	-2.612	1.387	0.060
TL	-1.392	0.886	0.116
TU	0.694	1.087	0.523
ST	-3.677	1.537	0.017
SN	-4.855	1.093	<0.001
Mean global RNFL	-3.086	0.864	<0.001

RNFL: retinal nerve fiber layer, SD: standard deviation, NU: nasoupper, NL: nasolower, IN: inferonasal, IT: inferotemporal, TL: temporolower, TU: temporoupper, ST: superotemporal, SN: superonasal;

Multivariate linear mixed models with adjustment for axial length.

## Discussion

Normal RNFL thickness variability has been evaluated in terms of race, age, sex, and axial length using SD-OCT technology. To our knowledge, the current report is the first to present data from the normal elderly population in Taiwan. We have provided normative, population-based RNFL thickness data from elderly people living in a rural area (Puzih City) in Taiwan, and clarified how RNFL thickness relates to both age and axial length.

When designing the study that evaluates the change of RNFL thickness in normative elderly people, any participants with ocular or non-ocular diseases that affect the RNFL thickness must be excluded. It is known that the retina forms as a functional extension of the central nervous system, developing as a result of diencephalic evagination of pluripotent cells during embryogenesis [15]. In many cross-sectional studies, the RNFL has been shown to be thinner in people with memory loss and cognitive decline due to certain neurodegenerative disorders, such as AD and MS [6–9]. There is also some controversy regarding the effect of lens opacity on signal detection in OCT. Therefore, we only included subjects with mild to moderate cataracts and without AD, MS, or other cognitive impairment, thus excluding any potential bias caused by dense cataracts, the effects of cataract extraction, or other neurodegenerative disorders. Furthermore, one strong point of our data is that the participants were not selected from a clinical ophthalmologist’s practice, but from a large, population-based, cohort study that randomly included 82 participants (143 eyes) over 65 years old from a general population (6786 village residents aged ≥ 65 years). However, our study also had two major limitations. First, the sample size of our study was small and the number of participants over the age of 80 was particularly low (n = 8). It was not easy to gather elderly people (age > 80 years) with normal eye structures that were not affected by other diseases or ocular surgical procedures. The second limitation, there was a potential for selection bias because all of our participants were volunteers. Therefore, we chose the statistical method, generalized estimating equation (GEE), to make each eye from a participant with same age represented a meaningful sample. In a randomized cross-sectional study, by such a sampling method, these 82 participants (143 eyes) can represent and make sense to a population in statistics. In addition, the negative linear correlation had been also confirmed by Pearson correlation coefficient (Pearson’s r = -0.210, p = 0.006) in our study, which also revealed the reverse relationship between aging in elderly and RNFL thickness (data not show). We also analyzed another 64 eyes (12 eyes in age < 70, 44 eyes in age between 70 and 79, and 8 eyes in age > 80) with post-cataract surgery (Table 1). In total 207 eyes (143 with non-cataract surgery and 64 with post-cataract surgery), the same trade was also significantly presented (P: 0.048, data not showed). So we confirmed that our sampling and analysis of data was meaningful.

The mean global peripapillary RNFL thickness in our study population was 102.84 ± 9.21 μm, which showed higher than previously published normative measurements involving a similar age group. For example, in one study involving 53 subjects aged 60 to 79 years, the mean RNFL thickness measured by SD-OCT was 89.6 ± 4.73 μm [16]. Another investigation that enrolled 210 subjects aged ≥ 75 years found a global RNFL thickness of 91.4 ± 12.6 μm [17]. Ethnic variations in RNFL thickness measured by OCT have been reported in normal subjects, and people of European descents tend to have a thinner RNFL than subjects of other ethnicities [18, 19]. In a study using Spectralis^™^ SD-OCT, Alasil et al. reported a mean RNFL thickness of 96 μm in subjects of European descent, which was significantly lower than the equivalent measurements in participants of Hispanic (102.9 μm) or Asian (100.7 μm) heritage [19]. Likewise, our data revealed a difference between those of Taiwanese and European descent in terms of mean RNFL thickness in the elderly. Conversely, we corroborated the data from Alasil et al. concerning the Asian population, despite using a different SD-OCT model (Zeiss, Stratus OCT 3000 vs. Spectralis SD-OCT).

In our data, the RNFL was thickest in the inferotemporal segment, followed by the superotemporal segment; the thinnest RNFL occurred in the nasal and temporal segments. The result followed the classic “ISNT” rule that characterizes the normal neuroretinal rim as reported in previous studies [19, 20]. This was also consistent with the characteristic configuration of the normal optic disc and cup shape, whereby the disc is a vertical oval and the cup is more horizontal. We concluded that the ISNT rule applies to the morphology of RNFL in many normal eyes among the elderly in Taiwan.

Regarding the relationship between RNFL thickness and age, various studies have been conducted and each reported varying results. No significant observation of RNFL thinning with age was found. Similar results were reported by Pakravan et al. (cross-sectional observational study of 96 normal Iranian subjects 20–53 years old) and Vernon (cross-sectional observational study of 31 highly myopic participants of European descent) [21, 22]. They hypothesized that a large variation (7 × 10^5^ to 1.4 × 10^6^ fibers) exists in the retinal ganglion cell complex in the normal population, which complicates the analysis [21]. The non-randomized study by Dhami et al. included 298 eyes of 149 healthy northern India individuals aged 10–70 years. The study suggested that the average RNFL thickness measured by Fourier domain OCT (RTVue-100, Optovue, Fremont, CA) does not decrease with age, but shows an inverse correlation with axial length [23]. A total of 4648 eyes in 2324 normal, randomly-selected Chinese students aged 6 to 17 years was examined in another study conducted by Chen et al.; they concluded that the RNFL thickness values were not correlated with age but with axial length [24]. These studies did not focus on the elderly population, reflecting that the RNFL thickness would not change significantly with age in younger subjects.

Contradictory results were observed by other researchers. They demonstrated that the average RNFL thickness decreases with age, and suggested that the reason was the probable loss of large numbers of axons with increasing age. Alasil et al. reported that RNFL thickness decreases by approximately 0.18% per year, which constitutes an average of 2 μm (range: 1.8–2.4 μm) per decade in people of European descent [19]. Khawaja’s EPIC-Norfolk Eye Study included 6309 subjects (11030 eyes) with a mean age of 68 years (range: 48–90 years) and showed a 1.53 μm decline per decade in British adults [25]. Regarding the rate of RNFL thickness decline with age in our study, as shown by multivariate analysis in Table 3, the global RNFL thickness measured by OCT decreased by 4.97 μm per decade (β = -0.497 [0.497 μm annually]). This result constitutes a steeper decline than those found in Alasil’s and Khawaja’s studies. In addition, the negative linear correlation had been also identified by Pearson correlation coefficient (Pearson’s r = -0.210, p = 0.006) in our study, which also revealed the reverse relationship between aging and RNFL thickness (data not show). Based on the evidence, we believe that the rate of RNFL thickness decline with age in normal elderly populations varies among ethnicities.

In the current study, age-related RNFL thickness decline was obvious in the superior and inferior halves, but the nasal and temporal halves did not differ in this regard. Notably, age-related RNFL thickness decline is more apparent in the thickest RNFL segments [17, 18]. The main retinal blood vessels are located superotemporally and inferotemporally to the optic nerve, and it has been suggested that the greatest age-related RNFL thickness decline in normal retinas occurs where the main retinal blood vessels reside [26]. It is unclear why these two areas might be preferentially affected, but it may due to the fact that nerve fibers with larger diameter atrophy more rapidly and those fibers are more abundant in the superotemporal and inferotemporal quadrants [27].

When we divided the peripapillary neuroretinal rim into eight segments for analysis, the superonasal (p = 0.004), inferotemporal (p = 0.046), and temporolower (p = 0.009) segments showed statistical significance in terms of age-related RNFL thinning. The same trend occurred in the superotemporal segment, although without significance, but not in the other four segments (nasoupper, inferonasal, temporoupper, and nasolower).

In normal adults, the optic nerve has approximately 1.0–1.2 million ganglion cell axons, and humans lose approximately 7500 (0.625%) axons per year after the age of 50 [28]. In our study, the annual decline in global RNFL thickness was 0.483%, which is a smaller reduction than the theoretical value, but is similar. The similarity of these two estimates of age-related RNFL decline, 0.625% and 0.483%, validates the correlation between histological and OCT findings. Moreover, it may be that the percentage decrease of axons differs from that of RNFL thickness because the RNFL is not composed of axons alone.

In current studies, myopic eyes with longer axial lengths had thinner RNFL, as measured using time-domain and SD-OCT [19, 29, 30]. In our sturdy, we revealed that the mean RNFL thickness was significantly related to axial length. Kang et al. found that, in subjects with myopia, the RNFL thins preferentially at the superior and inferior poles, and with a temporal upward deviation in peak thickness [31]. Kim et al. reported that subjects with high myopia had significantly thinner mean RNFL in the non-temporal parapapillary sectors, but thicker RNFL in the temporal quadrant [29]. Leung et al. reported similar results, whereby the RNFL was significantly thinner with worsening myopia in all quadrants except for the temporal quadrant [30]. The same investigators suggested that elongation of the globe in myopic eyes leads to mechanical stretching and thinning of the retina. Interestingly, the RNFL in eyes with a longer axial length was thinner in the nasal segments than in temporal segments; the trend was most noticeable in the superonasal and inferonasal segments.

The main retinal blood vessels are in the superotemporal and inferotemporal sectors. We suggest that the relatively richer blood supply in the temporal area prevents damage to this sector in highly myopic eyes. Results for age-related RNFL changes are contrary; the RNFL most marked age-related decrease occurred in the superotemporal and inferotemporal areas.

Our data revealed that a thinner RNFL was correlated with increased axial length in all areas except the temporal side. We conclude that the highly myopic eye should be considered in differential diagnoses, especially if the RNFL is thicker in temporal segments and thinner in all others.

### Conclusions

Our study showed that in normal elderly Taiwanese individuals, RNFL thickness tends to decrease with age. This decline is statistically significant in the superonasal, inferotemporal, and temporolower segments. Furthermore, Taiwanese people differ from those of European descent in terms of normative RNFL thickness. Information regarding the relationship between RNFL thinning and normal aging is important for longitudinal follow-up of pathological RNFL thinning in diseased subjects. To qualify as pathological, RNFL thinning must be measurably greater than that of normal aging. Our data also showed that a thinner RNFL was correlated with increased axial length in all areas except the temporal side. Therefore, our study is important to establish normative RNFL values in elderly Taiwanese individuals and to distinguish between disease-related and normal RNFL changes.
